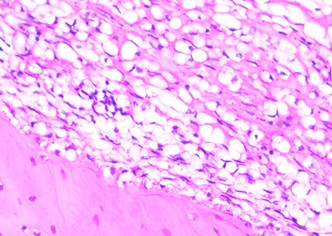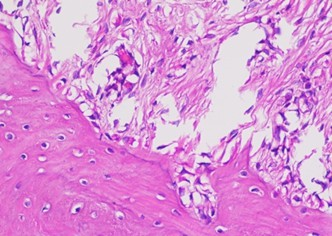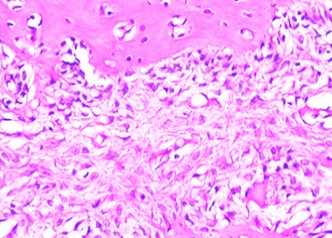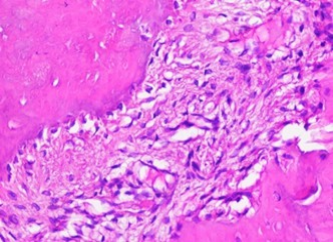# Expression of BMP-2, OPG, RANKL And Number of Osteoblasts After Application of Bovine Amniotic Membrane and Secretome Umbilical Cord in Wistar Rats

**DOI:** 10.7150/ntno.121118

**Published:** 2026-01-01

**Authors:** Ernie Maduratna Setiawatie, Lambang Bargowo, Shafira Kurnia Supandi, Vinanto Putero Negoro, Ferlina Diah Ayu Y. P. A., Elizabeth Luna K. A., Reinaldo Agusta, Nor Adinar Baharuddin

**Affiliations:** 1 Department of Periodontology, Faculty of Dental Medicine, Universitas Airlangga, Surabaya 60132, Indonesia.; 2 Periodontology Specialist Program, Faculty of Dental Medicine, Universitas Airlangga, Surabaya 60132, Indonesia.; 3 Department of Restorative Dentistry, Faculty of Dentistry, University of Malaya, Lembah Pantai, 50603 Kuala Lumpur, Malaysia.

**Keywords:** bovine amnion membrane, secretome, socket preservation, osteogenesis

## Abstract

**Introduction:** Bovine amniotic membrane (BAM) and secretome are tissue engineering materials studied for their high healing effects. This study aims to evaluate the expression levels of BMP-2, OPG, RANKL, and osteoblast count following the administration of BAM combined with secretome in Wistar rats undergoing socket preservation procedures.

**Method:** This study is a pure experimental study with a randomised post-test only control group design. Pre-test measurements were not performed because the baseline condition of bone healing cannot be assessed prior to extraction without interfering with socket healing. Randomization ensured that all groups started with equivalent baseline conditions, allowing reliable comparison of outcomes across groups. The expression of BMP-2, OPG, RANKL, and the number of osteoblasts in the dental tissues of male Wistar rats were examined post-extraction following socket preservation. The research variables consisted of control, as well as those treated with BAM, secretome, and BAM-secretome. One-Way ANOVA analysis and Tukey's Post-Hoc test were conducted to compare the expression of dependent variables between treatment groups.

**Results:** One-Way ANOVA and Post-Hoc Tukey tests showed that the expression of BMP-2, OPG, RANKL, and the number of osteoblasts in the BAM-secretome group were significantly higher compared to control, BAM, and secretome (p<0.001).

**Conclusion:** The combination of BAM with secretome significantly enhanced the expression of BMP-2, OPG, and osteoblast counts compared to BAM, secretome alone, and control groups with no treatment. These findings suggest that BAM-secretome holds promising potential for promoting bone regeneration. However, further phased clinical trials are essential to evaluate its safety and efficacy in socket preservation treatments.

## Introduction

Periodontal disease is one of the dental and oral health problems that has a high prevalence in Indonesia. Based on data from the Indonesian Basic Health Research (RISKESDAS) in 2018, 67.8% of people aged more than or equal to 15 years experienced periodontitis.^1^ Periodontitis is an infectious disease characterized by inflammation of the periodontal tissue.^2^ Bacteria such as *Porphyromonas gingivalis* found in dental plaque, will initiate inflammation of the gingiva, also known as gingivitis. When gingivitis is not treated properly, this condition can develop into periodontitis in susceptible individuals, where damage occurs to the periodontal ligament and alveolar bone. This condition can also be characterized by the formation of periodontal pockets and resorption of the alveolar bone, which if not treated immediately can cause various problems such as tooth loss and chewing dysfunction.^3^,^4^

Teeth with severe mobility due to periodontitis are typically extracted. Post-extraction, periodontal therapy may involve surgical procedures and dental implant placement. Implant placement can only be done if the condition of the alveolar bone is quite ideal. This can be achieved by performing socket preservation, a procedure that aims to maintain the horizontal and vertical dimensions of the edentulous ridge after tooth extraction.^5^,^6^ Cases that require socket preservation after extraction are determined based on aesthetic, functional and risk level considerations.^5^

According to several studies that have been done previously, materials commonly used in socket preservation include mesenchymal stem cells (MSCs), platelet-derived growth factor (PDGF), fibroblast growth factor, insulin growth factor, and bone morphogenetic proteins (BMPs). These materials have the ability to induce tissue regeneration processes. Determining the types of material is an important factor in the success of the socket preservation process.

Tissue engineering technology in dental medicine enhances healing by facilitating and guiding the regeneration process through targeted biological manipulation. In the tissue engineering approach, there are three important components, namely progenitor cells, conductive scaffolds, and signaling molecules.^8^ Progenitor cells have the ability to differentiate into various types of different adult cells.^8^ Scaffolds are three-dimensional structures that serve as a place for cells to proliferate, so that they can form tissues with the expected functions.^9^ Signaling molecules are defined as macromolecules that can be either proteins or lipids and function to provide signal to cells and induce growth.^10^

Bovine amnion membrane (BAM) is an alternative to the human amnion membrane as a scaffold for tissue stem cells. BAM has inhibitory power against bacterial colonization, contains stem cells, growth factors and proteins that can accelerate the regeneration process.^11^ In the epithelium, BAM has a function as stimulator of epithelialisation and differentiation of fibroblasts.^12^ Secretome is defined as a set of molecules and other biological components secreted by cells into the extracellular space. Secretome contains various serum proteins, growth factors, angiogenic factors, hormones, cytokines, extracellular matrix proteins, proteases, lipid mediators, and genetic materials, which makes many researchers develop it for tissue healing therapy^13^ (Lotfinia¸ et al., 2018).

The bone regeneration process occurs through osteogenesis which involves Bone Morphogenetic Proteins 2 (BMP-2) as a growth factor, OPG and RANKL.^14^ BMP-2 is a protein that stimulates the growth of osteoblasts through cellular pathways.^15^ RANKL has a function to activate osteoclasts, which play role in bone resorption. Excessive bone resorption will be prevented by OPG, by binding to RANKL thereby inhibiting osteoclast activation.^16^ This study aims to evaluate the bone regeneration process in Wistar rats with periodontitis following the administration of freeze-dried bovine amniotic membrane and secretome by assessing differences in the expression of Bone Morphogenetic Protein-2 (BMP-2), osteoprotegerin (OPG), receptor activator of nuclear factor kappa-Β ligand (RANKL), and osteoblasts.

## Research Methods

The study protocol was reviewed and approved by the Ethics Committee of the Faculty of Dental Medicine, Universitas Airlangga, Surabaya, Indonesia (Ethical Clearance No: 0498/HRECC.FODM/V/2024). All procedures involving experimental animals were conducted in accordance with institutional and international guidelines for animal care and use. A total of 56 male Wistar rats, aged 3 months and weighing about 150 - 300 g, were used as animal models in this study. All Wistar rat samples were locally induced by injecting 0.03 ml of *Porphyromonas gingivalis* bacteria at a concentration of 2 × 10⁶ CFU/ml from the facial direction into the mesial gingival sulcus of the left mandibular incisors. The procedure was repeated every 3 days for 2 weeks to establish periodontitis conditions in the Wistar rats. The rats were randomly divided into 8 groups, with each group consisting of seven rats. Group K served as the experimental control group, Group P1 received BAM, Group P2 was treated with hUC-MSCs secretome, and Group P3 received a combination of BAM and hUC-MSCs secretome. Each group was further subdivided for observations on day 7 and day 14 post extraction. Day 7 represents the early proliferative phase of bone healing, characterized by angiogenesis, inflammatory cell activity, and initial osteoblast recruitment. Day 14 corresponds to the early bone formation stage, where new woven bone begins to form and remodeling activity becomes more evident. These time points were chosen to capture the critical early stages of socket healing and regeneration.

The mandibular left incisor of each Wistar rat was extracted following standard protocols for handling experimental animals. The mandibular incisor tooth of Wistar rats was selected because of its large single-root morphology, ease of extraction, and accessibility for histological analysis. The Wistar rats were given intramuscular anesthesia in the femoral region with a dose of xylazine and ketamine given at 0.1 ml/10 grams of rat body weight. The dose prepared from a mixture of 1 ml of 100 mg/ml ketamine and 0.5 ml of 20 mg/ml xylazine.^17^ Irrigation using saline solution was performed on the socket to ensure that no granulation tissue was left behind. 18

In Group P1, BAM was applied into the tooth socket using an excavator. In Group P2, 0.3 ml of hUC-MSCs secretome was dispensed into the socket using a micropipette. Meanwhile, in Group P3, BAM mixed with secretome was rolled and inserted into the socket. The socket was then sutured using polyamide monofilament thread. On days 7 and 14, the experimental animals were sacrificed using 10% ether inhalation anesthesia. The mandible of the Wistar rats were harvested by cutting with a small saw and fixed in 70% formalin solution.

The mandibles of Wistar rats were decalcified in 10% EDTA solution for one month, with daily solution replacement, followed tissue processing for the histological preparation. The mandible tissue was embedded in paraffin using a block mold, frozen, sectioned and mounted on glass slides for observation. Quantitative observations of BMP-2, OPG and RANKL expression were performed using immunohistochemical (IHC) methods under a 400x magnification light microscope across 5 fields of view. Similarly, Meanwhile, osteoblasts were quantitatively observed using hematoxylin and eosin (HE) staining technique, also under a 400x magnification light microscope across 5 fields of view. The collected data were analysed using an image tool and recorded accordingly. This approach utilises the antigen-antibody reaction principles to determine the localization of target protein within tissues or cells.

## Results

### Research data

This study measured BMP-2, OPG, RANKL expression, and osteoblast count to assess bone regeneration after administration of BAM and secretome. The mean (x) and standard deviation (SD) values for each group are listed in the table (Table 1) based on the research findings.

Table 2 presents the results of the Kolmogorov-Smirnov test, which was used to assess whether the distribution of data for osteoblast numbers and the expression levels of BMP-2, OPG, and RANKL followed a normal distribution. The data were tested across four different groups—Control, BAM (Bovine Amniotic Membrane), Secretome, and BAM-Secretome—at two time points: day 7 and day 14. All the p-values obtained from this test were greater than 0.05, with most reaching the upper reporting limit of 0.200. This indicates that there was no significant deviation from a normal distribution for any of the measured variables across all groups and time points. Therefore, the assumption of normality is met, allowing the use of parametric statistical analyses in subsequent testing.

Meanwhile, Table 3 summarizes the results of Levene's test, which was conducted to evaluate the homogeneity of variances among the different treatment groups. This test was performed separately for each variable—BMP-2, OPG, RANKL, and osteoblast number—at day 7 and day 14. The results show that all p-values were above the 0.05 threshold, suggesting that the variances between groups were statistically similar. In other words, there was no significant difference in variance for any of the variables across the different experimental groups. This finding confirms that the data satisfy the assumption of equal variance, which is another key requirement for conducting parametric tests like ANOVA. Altogether, the results from both tables indicate that the dataset meets the assumptions for valid parametric analysis.

One-Way Anova test showed that there were differences in the expression of BMP-2, OPG, RANKL, and the number of osteoblasts in the control, BAM, secretome, and BAM-secretome groups (p<0.001) as shown at Table 4. The significant difference was found in post-extraction samples on day 7 and day 14.

Table 5 presents the results of Tukey's post hoc analysis comparing the expression of BMP-2 among the four groups—Control, BAM, Secretome, and BAM-Secretome—on day 7. The most significant differences were observed between the BAM-Secretome group and all other groups, with p-values less than 0.001, indicating highly significant increases in BMP-2 expression in the BAM-Secretome group. Meanwhile, no significant differences were found between the Control, BAM, and Secretome groups when compared pairwise, as shown by p-values well above 0.05. These results suggest that the combination of BAM and Secretome yields a synergistic effect on BMP-2 expression by day 7.

Table 6 displays pairwise comparisons of BMP-2 expression among groups on day 14. Similar to day 7, the BAM-Secretome group demonstrated significantly higher BMP-2 expression compared to the other groups (p < 0.001). However, the differences between Control, BAM, and Secretome were not statistically significant, indicating that the significant elevation in BMP-2 expression remains specific to the BAM-Secretome combination. This reinforces the potential long-term effect of the combined treatment in enhancing BMP-2 expression.

Table 7 illustrates the comparative analysis of OPG expression between treatment groups on day 7. A highly significant increase in OPG expression was observed in the BAM-Secretome group compared to the other three groups (p < 0.001). In contrast, no statistically significant differences were found between the Control, BAM, and Secretome groups, suggesting that only the combination treatment significantly stimulates OPG expression in the early phase of healing.

On day 14, the BAM-Secretome group continued to show a statistically significant increase in OPG expression relative to all other groups, with p-values below 0.001 (Table 8). The differences among the Control, BAM, and Secretome groups remained statistically insignificant. This consistent pattern further confirms the enhanced osteogenic environment promoted by the BAM-Secretome treatment over time, particularly in regulating OPG as an important factor in bone remodeling.

Table 9 shows significant reductions in RANKL expression in the BAM-Secretome group when compared with Control, BAM, and Secretome groups on day 7 (p < 0.001). No significant differences were detected among the other groups. Given that RANKL is a promoter of bone resorption, this significant decrease suggests a beneficial effect of the BAM-Secretome treatment in reducing bone resorption activity during the early phase of healing.

By day 14, the BAM-Secretome group maintained significantly lower RANKL expression compared to all other groups (p < 0.001) as shown at Table 10. Interestingly, while some non-significant differences were observed among the other groups, the BAM-Secretome treatment continued to provide a distinct suppression of RANKL expression. This suggests that the anti-resorptive effect of the combined treatment is sustained over time, supporting improved bone healing outcomes.

In Table 11, the number of osteoblasts was significantly higher in the BAM-Secretome group compared to Control, BAM, and Secretome groups on day 7 (p < 0.001). No significant differences were noted among the other groups, with p-values exceeding 0.05. This finding indicates that the BAM-Secretome combination not only enhances molecular markers of bone formation but also effectively increases the population of osteoblasts, suggesting a robust regenerative response in the treated group.

The Tukey Post-Hoc test showed differences between the two groups of independent variables as shown at Table 12. Significant differences were obtained in the test results with a p value < 0.05. The expression of BMP-2, OPG, RANKL and the number of osteoblasts showed significant values in all comparisons between BAM-secretome and other groups, both on day 7 and day 14. Positive values in Mean differences indicate significant differences in the expression of BMP-2, OPG, RANKL and the number of osteoblasts which were higher in BAM-secretome compared to other groups.

### Immunohistochemical Examination Result

Immunohistochemical staining technique was performed on bone tissue preparations using monoclonal antibodies anti BMP-2, anti OPG, and anti RANKL.

The Table 13 above showed the results of histological observations of BMP-2, OPG, and RANKL expression in socket area after tooth extraction. Observations were made using a light microscope with 400x magnification. BMP-2, OPG, and RANKL expression were seen in all groups, both on day 7 and day 14.

### Hematoxylin and Eosin Examination Result

Hematoxylin Eosin (HE) staining was performed on bone tissue preparations to see the presence of osteoblast cells and to calculate the number of osteoblast cells.

The table above showed the results of histological observations of osteoblast expression in socket area after tooth extraction. Observations were made using a light microscope with 400x magnification. Osteoblast expression was seen in all groups, both on day 7 and day 14.

## Discussion

Socket preservation is a technique used to manage the residual alveolar bone post-extraction, aiming to reduce bone loss and maintain ridge dimensions and density, so that the area where the tooth has been extracted is suitable for dental implant placement. Socket preservation can be done through a tissue engineering approach, by adding graft or scaffold material into the tooth socket.^19^,^20^,^5^

The presence of osteoblasts, BMP-2, OPG, and RANKL, as part of the osteogenesis process, greatly influences the success of socket preservation.^20^ This study applies tissue engineering as one of the socket preservation methods by assessing BMP-2, OPG, RANKL and the number of osteoblasts in Wistar rat teeth after extraction on the 7th and 14th days.

The findings showed that BMP-2 expression increased significantly in all experimental groups compared to control, with the BAM-secretome combination showing the highest levels on both days 7 and 14. BMP-2, a key osteogenic factor, plays an important role in initiating osteoblast differentiation and bone formation. The increased BMP-2 in the BAM-secretome group suggests a synergistic effect between the scaffold (BAM) and the bioactive secretome. The synergistic effect observed in the BAM-secretome group may be explained by the complementary biological roles of both components. The bovine amniotic membrane acts as a biocompatible scaffold that supports cell attachment, proliferation, and differentiation. It contains extracellular matrix proteins and intrinsic growth factors that stimulate fibroblast activity and angiogenesis. The secretome, derived from hUC-MSCs, contributes a rich mixture of cytokines, chemokines, angiogenic factors, and extracellular vesicles, which enhance osteoblast differentiation, stimulate vascular ingrowth, and modulate the local immune response. The interaction between BAM and secretome creates a microenvironment where BMP-2 upregulation promotes osteoblastogenesis, OPG counteracts RANKL-mediated osteoclast activation, and the balance shifts toward bone formation. This biological interplay explains the significantly higher osteoblast numbers and more favorable bone remodeling activity seen in the BAM-secretome group compared with other groups. Research conducted by Ariesta et al. (2023) found that the application of BAM alone can increase BMP-2 expression in alveolar bone socket preservation after the 14th and 28th days. These results are in line with this study.^24^

Similarly, OPG expression, which helps inhibit osteoclast differentiation by acting as a decoy receptor for RANKL, was also significantly elevated in the BAM-secretome group. This study found that the highest OPG expression after extraction on the 7th and 14th day was found in samples treated with BAM combined with secretome, compared to controls, BAM or secretome alone. These results were significant after being tested with the Post hoc test. High OPG expression triggers bone regeneration, which is important to support more effective socket preservation.^27^

The results of this study demonstrated that RANKL expression in the socket preservation sites of Wistar rat teeth was significantly higher in the group treated with a combination of BAM and secretome on both day 7 and day 14 post-extraction. This finding was confirmed through Tukey post hoc analysis, which showed a statistically significant difference compared to the control, BAM alone, and secretome alone groups. The elevated RANKL expression in this group may indicate active bone remodeling, which is crucial in the early stages of healing. While RANKL is known to stimulate osteoclast differentiation and bone resorption, its increased expression—when accompanied by high OPG levels as observed in this study—suggests a balanced regulation of bone turnover rather than pathological resorption. This aligns with the physiological role of RANKL and OPG in maintaining bone homeostasis. These findings support the hypothesis that the BAM-secretome combination promotes a controlled bone remodeling environment, facilitating optimal socket healing. This result is consistent with previous studies emphasizing the importance of RANKL in initiating remodeling while being modulated by OPG to prevent excessive bone loss.^27^,^26^

Osteoblast count, which directly indicates bone formation activity, was highest in the BAM-secretome group. This confirms the molecular findings and indicates that the combination treatment not only promotes signaling pathways for bone regeneration but also leads to active cellular participation in new bone deposition. The consistent increase from day 7 to day 14 suggests sustained regenerative stimulation over time. Wibowo et al. (2023) found that osteoblast expression was higher in BAM applications compared to controls in socket preservation.^30^ The presence of osteoblasts can accelerate bone healing along with increased osteocytes and collagen.^27^,^30^ This study found that osteoblast expression on the 7th and 14th day after extraction was highest in samples treated with a combination of BAM and secretome compared to controls, BAM, or secretome alone.

Systematic review has concluded that graft method is one of the best choices in alveolar ridge preservation because it can reduce alveolar bone resorption in the healing process after tooth extraction.^31^ Other studies have shown that bovine bone graft, which is derived from the same xenograft as BAM, shows higher expression of BMP-2, OPG, and RANKL compared to the control group. This condition can trigger bone growth more effectively.^31^,^32^,^33^,^34^

BAM, which is an amniotic membrane, has an effectiveness that is not significantly different from deproteinized bovine bone (DBB) in the application of socket preservation.^35^ A systematic review stated that the application of amniotic membranes such as BAM will be more efficient than bone grafts such as DBB because it has superior ectopic sites and orthotopic sites so that it can increase the number of osteoblasts faster, especially when applied together with secretome. Bone formation becomes more optimal along with the increase in the number of osteoblasts.^36^,^37^

This study added the hUC-MSCs secretome because it has been proven to have an important role in the development of oral regenerative medicine. Secretome has a biotherapeutic effect that triggers the proliferation and migration of MSCs and osteoprogenitor cells. Within 12 weeks, bone and connective tissue volume can increase significantly. Angiogenesis begins to occur in the 4th week. The three effects of the secretome can allow bone cell regeneration which can also create compatible socket preservation.^36^,^38^,^39^,^40^ This theory may explain the superiority, seen from the significantly lowest osteoclast expression in the combination treatment of BAM and hUC-MSCs secretome compared to the control, BAM alone, or secretome alone in this study.

The clinical implications of these findings are highly relevant for regenerative dentistry. Alveolar bone loss following tooth extraction remains a major challenge for implant placement and prosthetic rehabilitation. The demonstrated ability of BAM combined with secretome to enhance BMP-2 and OPG expression, suppress RANKL activity, and increase osteoblast formation suggests that this strategy could serve as a novel biomaterial for socket preservation and bone defect management. In patients with alveolar bone defects, the BAM-secretome combination has the potential to accelerate early healing, maintain ridge dimensions, and improve bone quality, thereby reducing the need for more invasive grafting procedures.

### Strengths and Limitations

This study effectively investigated the expression of BMP-2, OPG, RANKL, and osteoblasts in osteogenesis after administration of bovine amniotic membrane combined with secretome. However, no toxicity or preclinical tests were conducted to determine the safety of using BAM grafts combined with hUC-MSCs secretome. Additionally, there may be other possible factors that have not been considered that could influence the expression of biomarkers (BMP-2, OPG, RANKL) and osteoblast activity. Further research is needed to address potential sources of bias and to evaluate the safety and efficacy of socket preservation treatments.

## Conclusion

This study demonstrated that the combination of bovine amniotic membrane and hUC-MSCs secretome significantly enhances bone regeneration in extraction sockets of Wistar rats. A clear relationship was observed between BMP-2, OPG, RANKL expression, and the number of osteoblasts at both observation times. On day 7, the BAM-secretome group showed the highest BMP-2 and OPG expression accompanied by suppressed RANKL levels, which correlated with an increased number of osteoblasts. This indicates that early in healing, the combined treatment promotes osteoinduction while limiting osteoclast-mediated resorption, thereby facilitating osteoblast recruitment. By day 14, BMP-2 and OPG remained significantly elevated, RANKL expression continued to be downregulated, and osteoblast counts further increased compared to all other groups. These findings suggest a sustained osteogenic environment in which BMP-2 drives osteoblast differentiation, OPG prevents excessive RANKL-mediated resorption, and the resulting balance leads to active bone formation.

## Figures and Tables

**Table 1:** Mean expression of BMP-2, OPG, RANKL and osteoblasts counts in control, BAM, secretome, and BAM-secretome groups on day 7 and day 14.

Group		Mean ± SD			
		BMP-2	OPG	RANKL	Osteoblast
Control	day 7	2,286±1,113	2,714±1,113	2,286±1,113	3,857±1,345
	day 14	3,571±1,272	3,714±1,113	3,000±1,414	4,857±1,345
BAM	day 7	2,857±1,345	3,286±1,113	3,714±1,113	4,857±1,345
	day 14	4,286±1,113	5,429±1,272	3,429±1,272	6,571±1,512
Secretome	day 7	3,286±1,113	3,857±1,345	3,286±1,113	3,571±1,272
	day 14	4,143±1,345	5,000±2,000	4,714±1,496	7,000±1,732
BAM - secretome	day 7	8,286±1,704	9,000±1,414	7,429±1,272	9,571±1,512
	day 14	8,423±1,397	9,143±1,345	7,286±1,113	10,000±1,826

**Table 2:** Kolmogorov-Smirnov Test for Normality of Data Distribution

Group		Kolmogorov-Smirnov Normality Test (p-value)			
		Osteoblas	BMP-2	OPG	RANKL
Control	day 7	0,200	0,200	0,200	0,200
	day 14	0,200	0,200	0,200	0,200
BAM	day 7	0,200	0,200	0,200	0,200
	day 14	0,200	0,200	0,200	0,200
Secretome	day 7	0,200	0,200	0,200	0,200
	day 14	0,200	0,200	0,155	0,200
BAM - secretome	day 7	0,200	0,200	0,200	0,200
	day 14	0,200	0,200	0,200	0,200

Note: p > 0,05 (Normal Distribution)

**Table 3:** Levene's Homogeneity Test of BMP-2, OPG, RANKL Expression and Osteoblast Number

Group		*Levene Statistic*	Sig.
BMP-2	day 7	0,814	0,499
	day 14	0,091	0,964
OPG	day 7	0,231	0,874
	day 14	2,134	0,122
RANKL	day 7	0,156	0,925
	day 14	0,266	0,849
Osteoblast	day 7	0,130	0,941
	day 14	0,396	0,757

Note: p > 0,05 (Homogeneous)

**Table 4:** One Way Anova Test of BMP-2, OPG, RANKL Expression and Number of Osteoblast

Group	Termination	*Uji One-Way Anova*	
		F	Sig.
BMP-2	day 7	29,848	< 0,001
	day 14	21,144	< 0,001
OPG	day 7	26,527	< 0,001
	day 14	17,555	< 0,001
RANKL	day 7	37,333	< 0,001
	day 14	14,678	< 0,001
Osteoblast	day 7	29,032	< 0,001
	day 14	12,288	< 0,001

**Tabel 5:** Tukey Post Hoc Test of BMP-2 Expression on Day 7 Among Experimental Groups

	Group 1:	Control		BAM		*Secretome*		BAM-*Secretome*	
		*Mean Differences* ± SD	Sig.	*Mean Differences* ± SD	Sig.	*Mean Differences* ± SD	Sig.	*Mean Differences* ± SD	Sig.
Group 2:									
Control				0,571 ± 0,716	0,855	1,000 ± 0,716	0,514	6,000 ± 0,716	< 0,001**
BAM						0.429 ± 0,716	0,932	5.4286 ± 0,716	< 0.001**
*Secretome*								5,000 ± 0,716	< 0.001**
BAM-*Secretome*									

**Tabel 6:** Tukey Post Hoc Test of BMP-2 Expression on Day 14 Among Experimental Groups

	Group 1:	Control		BAM		*Secretome*		BAM-*Secretome*	
		*Mean Differences* ± SD	Sig.	*Mean Differences* ± SD	Sig.	*Mean Differences* ± SD	Sig.	*Mean Differences* ± SD	Sig.
Group 2:									
Control				0,714±0,688	0,729	0,571 ± 0,688	0,839	4,857±0,688	< 0,001**
BAM								4.143±0,688	< 0.001**
*Secretome*				0,143±0,688	0,997			4.286±0,688	< 0.001**
BAM-*Secretome*									

**Table 7:** Tukey Post Hoc Test of OPG Expression on Day 7 Among Experimental Groups

	Group 1:	Control		BAM		*Secretome*		BAM-*Secretome*	
		*Mean Differences* ± SD	Sig.	*Mean Differences* ± SD	Sig.	*Mean Differences* ± SD	Sig.	*Mean Differences* ± SD	Sig.
Group 2:									
Control				0,571 ± 0,670	0,829	1,142 ± 0,670	0,343	6,285 ± 0,670	< 0,001**
BAM						0,571 ± 0,670	0,829	5,714 ± 0,670	< 0.001**
*Secretome*								5,143 ± 0,670	< 0.001**
BAM-*Secretome*									

**Table 8:** Tukey Post Hoc Test of OPG Expression on Day 14 Among Experimental Groups

	Group 1:	Control		BAM		*Secretome*		BAM-*Secretome*	
		*Mean Differences* ± SD	Sig.	*Mean Differences* ± SD	Sig.	*Mean Differences* ± SD	Sig.	*Mean Differences* ± SD	Sig.
Group 2:									
Control				1,714 ± 0,787	0,158	1,286 ± 0,787	0,514	5,429 ± 0,787	< 0,001**
BAM								3,714 ± 0,787	< 0.001**
*Secretome*				0.428 ± 0,787	0,379			4,143 ± 0,787	< 0.001**
BAM-*Secretome*									

**Table 9:** Tukey Post Hoc Test of RANKL Expression on Day 7 Among Experimental Groups

	Group 1:	Control		BAM		*Secretome*		BAM-*Secretome*	
		*Mean Differences* ± SD	Sig.	*Mean Differences* ± SD	Sig.	*Mean Differences* ± SD	Sig.	*Mean Differences* ± SD	Sig.
Group 2:									
Control				1,429 ± 0,617	0,123	1,000 ± 0,617	0,387	5,143 ± 0,617	< 0,001**
BAM								3,714 ± 0,617	< 0.001**
*Secretome*				0,429 ± 0,617	0,898			4,143 ± 0,617	< 0.001**
BAM-*Secretome*									

**Table 10:** Tukey Post Hoc Test of RANKL Expression on Day 14 Among Experimental Groups

	Group 1:	Control		BAM		*Secretome*		BAM-*Secretome*	
		*Mean Differences* ± SD	Sig.	*Mean Differences* ± SD	Sig.	*Mean Differences* ± SD	Sig.	*Mean Differences* ± SD	Sig.
Group 2:									
Control				0,429 ± 0,712	0,930	1,714 ± 0,712	0,102	4,286 ± 0,712	< 0,001**
BAM						1,286 ± 0,712	0,295	3,857 ± 0,712	< 0.001**
*Secretome*								2,572 ± 0,712	< 0.001**
BAM-*Secretome*									

**Table 11:** Tukey Post Hoc Test of Osteoblast Count on Day 7 Among Experimental Groups

	Group 1:	Control		BAM		*Secretome*		BAM-*Secretome*	
		*Mean Differences* ± SD	Sig.	*Mean Differences* ± SD	Sig.	*Mean Differences* ± SD	Sig.	*Mean Differences* ± SD	Sig.
Group 2:									
Control				1,000 ± 0,733	0,533			5,714 ± 0,733	< 0,001**
BAM								4,714 ± 0,733	< 0.001**
*Secretome*		0,286 ± 0,733	0,979	1,286 ± 0,733	319			6,000 ± 0,733	< 0.001**
BAM-*Secretome*									

**Table 12:** Tukey Post Hoc Test for Osteoblast Count on Day 14 Among Experimental Groups

	Group 1:	Control		BAM		*Secretome*		BAM-*Secretome*	
		*Mean Differences* ± SD	Sig.	*Mean Differences* ± SD	Sig.	*Mean Differences* ± SD	Sig.	*Mean Differences* ± SD	Sig.
Group 2:									
Control				1,714±0,863	0,221	2,143±0,863	0,088	5,143±0,863	<0,001**
BAM						0,429±0,863	0,959	3,429±0,863	<0.001**
*Secretome*								3,000±0,863	<0.001**
BAM-*Secretome*									

**Table 13:** Histological Observations of BMP-2, OPG, and RANKL Expression at Day 7 and 14

Group		Control	BAM	*Secretome*	BAM-*Secretome*
BMP-2	day 7	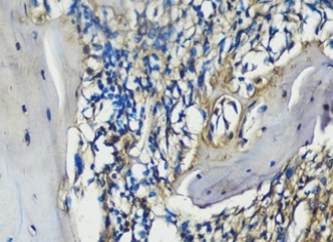	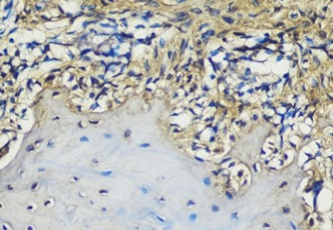	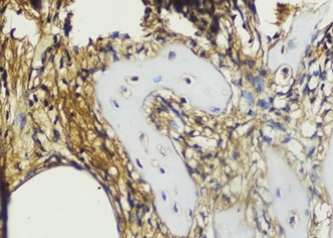	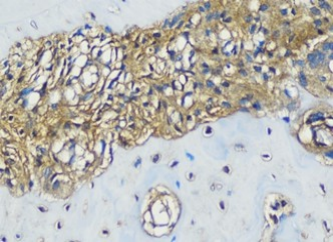
	day 14	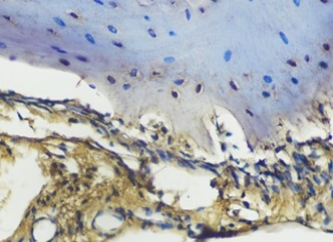	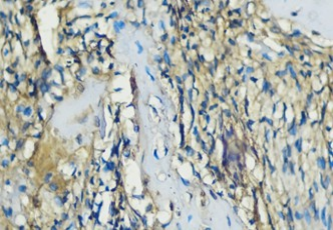	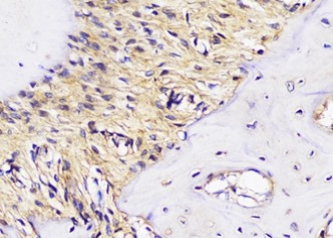	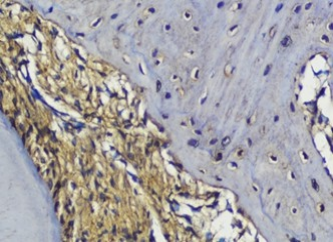
OPG	day 7	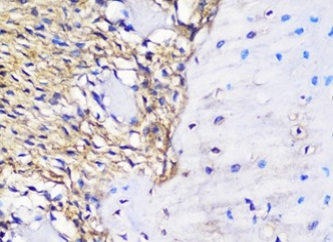	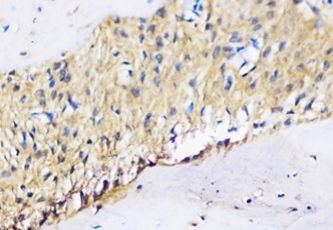	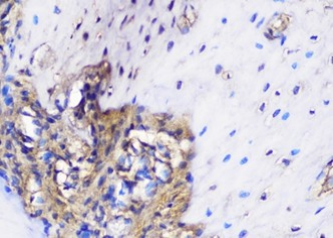	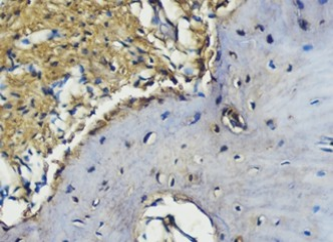
	day 14	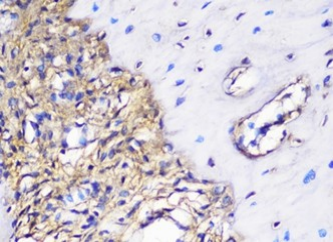	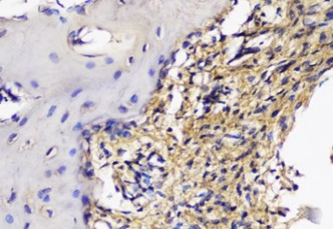	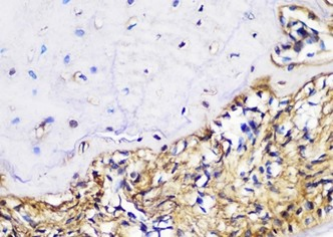	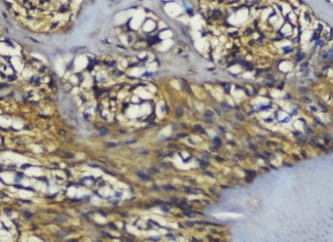
RANKL	day 7	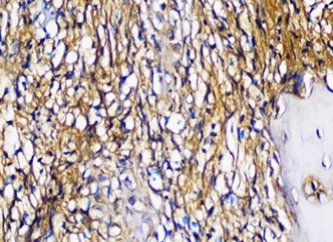	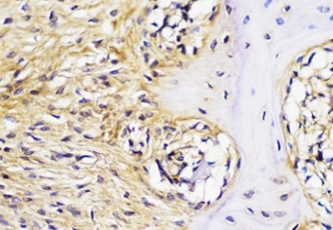	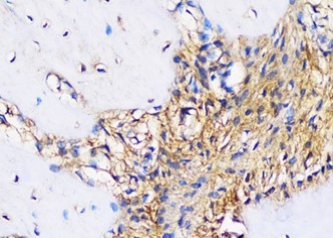	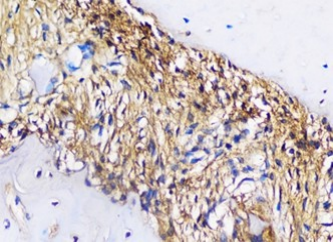
	day 14	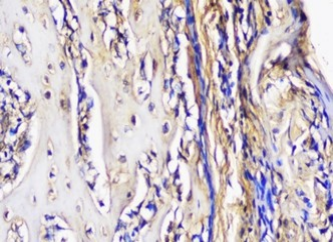	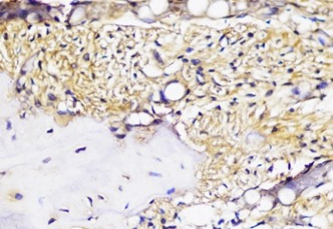	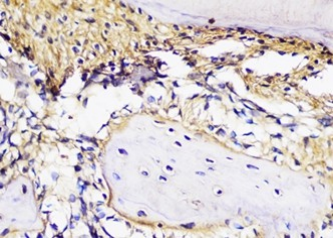	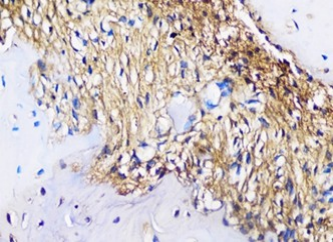

**Table 14:** Histological Observations of Osteoblast Expression at Day 7 and 14

Osteoblast	Control	BAM	*Secretome*	BAM-*Secretome*
Day 7	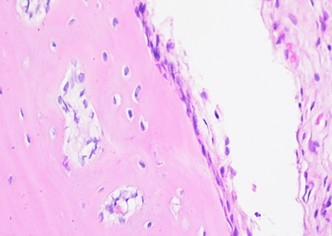	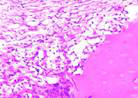	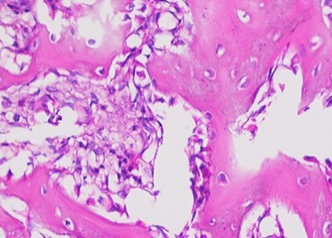	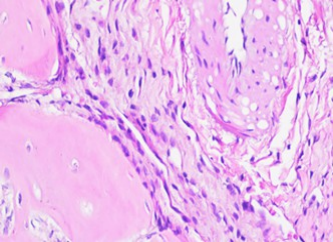
Day 14